# Digital Being: social media and the predictive mind

**DOI:** 10.1093/nc/niae008

**Published:** 2024-03-18

**Authors:** Ben White, Andy Clark, Mark Miller

**Affiliations:** School of Media, Arts and Humanities, University of Sussex, Arts A07, Brighton BN1 9RH, United Kingdom; School of Media, Arts and Humanities, University of Sussex, Arts A07, Brighton BN1 9RH, United Kingdom; Department of Philosophy, Macquarie University, Macquarie University Wallumattagal Campus Macquarie Park, Sydney, NSW 2109, Australia; Monash Centre for Consciousness and Contemplative Studies, Monash University, Wellington Rd, Clayton, Melbourne, VIC 3800, Australia; Psychology Department, University of Toronto, 100 St. George Street, 4th Floor, Sidney Smith Hall, Toronto, ON M5S 3G3, Canada

**Keywords:** active inference, social media, mental health, addiction, depression

## Abstract

Social media is implicated today in an array of mental health concerns. While concerns around social media have become mainstream, little is known about the specific cognitive mechanisms underlying the correlations seen in these studies or why we find it so hard to stop engaging with these platforms when things obviously begin to deteriorate for us. New advances in computational neuroscience, however, are now poised to shed light on this matter. In this paper, we approach the phenomenon of social media addiction through the lens of the active inference framework. According to this framework, predictive agents like us use a ‘generative model’ of the world to predict our own incoming sense data and act to minimize any discrepancy between the prediction and incoming signal (prediction error). In order to live well and be able to act effectively to minimize prediction error, it is vital that agents like us have a generative model, which not only accurately reflects the regularities of our complex environment but is also flexible and dynamic and able to stay accurate in volatile and turbulent circumstances. In this paper, we propose that some social media platforms are a spectacularly effective way of warping an agent’s generative model and of arresting the model’s ability to flexibly track and adapt to changes in the environment. We go on to investigate cases of digital tech, which do not have these adverse effects and suggest—based on the active inference framework—some ways to understand why some forms of digital technology pose these risks, while others do not.

## Introduction

A recent survey of young people online found that 48% had felt influenced or pressured by social media to consider having a cosmetic surgery procedure (Arab et al. 2019). Moreover, some specific social media influencers have spoken openly about how they undergo cosmetic procedures in order to ‘attract an audience online’, and to maximize the amount of positive engagement a post might receive (Truly 2019). While cosmetic surgery should not be thought of as intrinsically problematic, the potential link between social media platforms and a desire to undergo cosmetic surgery procedures has been dubbed ‘Snapchat surgery’ and represents the most recent addition to a litany of worries around social media, mental health, body image, and general wellbeing.

Today, social media is implicated in an array of mental health concerns. A 2017 report published by a parliamentary group in the UK linked social media use with a range of worries about mental health (Cramer and Inkster 2017), while a growing number of empirical studies link social media use with symptoms of addiction and depression (Lin et al. 2016, Andreassen et al. 2017). Worries that social media platforms might in some way warp our perception of the world, or cause low self-esteem or diminished life satisfaction, seem to be running through the mainstream collective psyche and even some former influencers have begun to turn against various social media platforms, highlighting the dangers of curating a self-image with little purchase on reality (Gritters 2019). In response, some platforms have begun trialling design tweaks aimed at protecting the user’s health, such as limiting the visibility of ‘likes’ on a post. Even national governments are beginning to trial new legislation, such as a proposed law in Norway that would require influencers to explicitly label which pictures have been digitally altered (Grant 2021).

While concerns around social media have become mainstream, little is known about the specific cognitive mechanisms underlying the purported correlations highlighted by these studies, and why some of us find it so hard to stop engaging with these platforms when things obviously begin to deteriorate. In what follows, we suggest that both the rise in Snapchat surgery, and the connections between social media, depression, and addiction, can be accounted for via a unified theoretical approach grounded in an emerging, and now highly influential, theory of cognition and affect—the active inference framework (AIF). We propose that the structure of some social media platforms constitute ‘hyperstimulating’ digital environments, wherein the design features and functional architecture of digital environments impact the machinery of cognition in ways which can lead to a warping of healthy agent-environment dynamics, producing precisely the sorts of pathological outcomes we see emerging today (Wilson 2014). In what follows, we will first explore some of the empirical literature on social media and mental health, including two recent reviews. We highlight how the empirical literature lacks consensus and has been the subject of criticism. We also reflect on how the state of the empirical literature motivates the need for the application of a good theoretical framework. Next, we will briefly introduce the AIF. In the third section, we highlight how the same predictive mechanisms that keep us alive and well can also become warped, leading to aberrant feedback loops in cognition and behaviour that help explain the various psychopathologies allegedly related today to social media and other digital online platforms. We then argue that various digital environments, including some social media platforms, have very specific design features and mechanisms that leave our predictive systems particularly vulnerable to these kinds of suboptimal feedback loops. In the final section, we briefly raise the puzzle of cases in which our interactions with online digital platforms do not have these negative outcomes, and speculate about some possible reasons why these cases might be different.

## Existing literature and motivation

Before committing to a detailed application of the AIF to thinking about social media, it is important to briefly review the empirical literature and reflect on the need for a deeper theoretical understanding. While the empirical research looking at the potentially detrimental outcomes associated with social media has failed to reach any kind of meaningful consensus, ourselves and other researchers believe that this highlights the critical need for a solid and consistent theoretical approach. In this section, then, we briefly survey the current state of social media research by looking at several different studies and recent reviews, and then reflect on the motivations for a theoretical approach.

Numerous studies have investigated possible links between using social media and various forms of reduced psychological well-being, including depression (Lin et al. 2016, Twenge 2017), addiction (Andreassen 2015, Longstreet and Brooks 2017), and anxiety (Vannucci et al. 2017). In fact, the volume of research, which finds correlations between social media use and forms of reduced psychological wellbeing, has resulted in the view that social media is harmful entering the mainstream. Jean Twenge, a psychologist investigating the relationship between teens and their technology, has published widely, arguing that smartphone use and social media may have ‘destroyed a generation’ (Twenge 2017). Similarly, former Silicon Valley workers have published books with titles like ‘Ten Arguments for Deleting your Social Media Accounts Right Now’ (Lanier 2018).

However, despite the purported links between social media and forms of reduced psychological wellbeing, some researchers have pushed back against claims that social media causes conditions like depression and anxiety. A recent study by Orben et al. (2019), for example, found that the relationship between social media use and reduced well-being was highly questionable, wherein the observed effects were small, nuanced, specific to gender, and highly contingent on the different analytic approaches applied (Orben et al. 2019). While we do not have space to perform our own systematic review here, other reviews have questioned and criticized much of the research on social media and mental health. A 2020 review by Amy Orben found that the effect sizes of these correlations (between social media and negative psychological well-being) are invariably very small, and strongly emphasized the important role played by a variety of other variables (Orben 2020a). Both Orben’s review and another review undertaken by Frost and Rickwood (2017) found that much of the research that claims to have found a casual relationship between social media use and reduced psychological wellbeing is of ‘poor quality’. The lack of quality is reflected in weak evidence, suboptimal measures such as general ‘screen time’, lack of transparency, lack of theoretical framework, poor participant cooperation, and other methodological issues. A 2022 review by Ghai *et al*. also pointed out, correctly, that the overwhelming majority of research into social media and mental health has neglected the Global South, thus underemphasising the role played by diverse cultural contexts in mediating the effects of social media use (Ghai et al. 2022).

Given the state of the empirical literature on the effects of social media, with its ‘poor quality’ studies and mixed findings, it might be argued that there is little motivation to perform a theoretical exploration of the purported negative effects. However, in applying the AIF to social media in this way, our aim is not to assume that these effects do exist but rather to show that the AIF is an apt and useful framework for explaining how such effects might be underpinned by specific cognitive and affective dynamics. Such an approach is warranted, for three reasons: first, despite the state of the empirical literature, there exists an overwhelming volume of anecdotal evidence and subjective narrative reporting that speaks to people’s experience of social media and the internet more generally, and perceived connections to reduced psychological wellbeing (See e.g. Wilson 2014, Gritters 2019). Second, we know that some social media platforms are deliberately designed to be addictive (Montag et al. 2019). Finally, empirical researchers working on social media and mental health have explicitly stated that many of the shortcomings in the current research programme are the result of a lack of theoretical framework (Orben 2020b). To be clear, our primary aim is to remedy this lack of theoretical approach and to demonstrate the power and flexibility of the AIF as an overarching framework for studying human technology and mental health.

## Introducing active inference

The revolutionary move of the AIF is to reimagine the brain as a prediction engine constantly attempting to predict the sensory signals it encounters in the world and minimizing the discrepancy (‘prediction errors’) between those predictions and the incoming signal (Friston 2010, Howhy 2013, Clark 2016). In order to make apt predictions, these systems need to build-up a ‘generative model’: a structured understanding of the statistical regularities in our environment, which are used to generate predictions. This generative model is essentially a model of our world, including both immediate, task-specific information, as well as longer-term information that constitutes our narrative sense of self. According to this framework, predictive systems can go about minimizing prediction errors in two ways: either they update the generative model to more accurately reflect the world, or they behave in ways that bring the world better in line with their prediction (Clark 2016). In this way, the brain forms a part of an embodied predictive system, which is always striving to move from uncertainty to certainty (Nave et al. 2020). By successfully minimizing potentially harmful surprises, these systems keep us alive and well. Consider the healthy and highly expected body temperature for a human being of 37°C. A shift in temperature in either direction registers as a spike in prediction error, signalling to the organism that it is moving into an unexpected, and therefore a potentially dangerous state. As long as the change in temperature is not too extreme, we could just sit there and come to terms with the changing temperature (update our generative model), or we might reach for a blanket or open a window. In these cases, what we are doing is acting upon our environment, sampling the world and changing our relation to it, in order to bring ourselves back within acceptable bounds of uncertainty.

Predictive systems must be flexible and able to quickly adapt to changing conditions within an environment. According to the AIF, the predictive system is flexibly tuned by second-order predictions that estimate the salience and reliability of the error units resulting from first-order predictions given the current context (Parr and Friston 2017). The so-called ‘precision weighting’ acts to modulate the impact that particular prediction errors have on the system. For example, high precision can drive learning and further processing, while low precision would render a signal relatively impotent within the system (Clark 2017). This mechanism allows the system contextual flexibility and can also allow greater reliance on either the generative model or sensory signals. Precision also plays a central role in selecting which behaviours are enacted, as actions are selected on the basis of expectations about future error reduction. That is to say, predictive agents score and select behaviours based on predictions about the likely error-reducing capacities of those behaviours within a given context. In other words, precision is weighted on beliefs about policies, given the likelihood that in a given context, a certain policy will lead to a certain reduction in error (Friston et al. 2017).

Crucial for this process is the sensitivity about how well we are managing error over time relative to expectations. ‘Error dynamics’ refers to changes in the rate of average error reduction over time (Joffily et al. 2013, Kiverstein et al. 2017, Van de Cruys 2017, Hesp et al. 2021). The rate of change in error can be visualized as a ‘slope’, with steep decreases in error minimization representing that the system is doing well at confirming predictions, and a steep increase as a loss of predictive acuity. On the agent level, changes in error dynamics are experienced as valenced bodily affect (i.e. positive and negative feelings accompanied by approach or avoidance tendencies). When an organism registers a slope of error reduction in line with (or better than) its expectations, they are ‘rewarded’ with positively valenced affective changes. When the rate of prediction error rises (or the rate of reduction slows down), the organism is ‘punished’ with a negatively valenced affect (Eldar et al. 2016). These affective changes play a role in the AIF by tuning precision weighting on action policies. Positive or negative valence acts as feedback to the system, up-regulating or down-regulating precision expectations, respectively. In short, valenced bodily affect shifts us toward a closer attunement with the environment, by raising or lowering the system’s confidence in sets of action policies relative to how well or poorly those behaviours have proven themselves to be relative to expectations (Kiverstein et al. 2020).

This means that as predictive organisms, we actively seek out waves of manageable prediction error—manageable uncertainty—because resolving it results in our feeling good. Of course, just how much uncertainty an agent finds manageable will vary from person to person. Predictive organisms that are tuned by error dynamics then will naturally exhibit curiosity and exploratory behaviour (Kiverstein et al. 2017). They will be moved affectively to seek out and make the most of the steepest slopes of error reduction in their environment. Situations which offer too little resolvable error (i.e. are too predictable) are boring for such organisms, while situations with too much error (i.e. too uncertain) are experienced by the agent as frustrating or threatening. The recent rise in jigsaw puzzle sales during the covid lockdown testifies to our love of manageable uncertainty. These feelings evolved to keep us well-tuned to our environment, helping us to curiously feel out novel and successful strategies for survival, while also avoiding all of the stress and unpleasantness which comes with runaway uncertainty. This active, recursive, and felt relationship with the environment is crucial to grasping how social media can be detrimental to our mental health, and why we often find it so hard to stop using it, as we will see next.

Living well, in active inference terms, means being able to effectively manage uncertainty over time—and that is predicated on having a generative model which represents the world accurately. A generative model that poorly reflects the regularities of the environment would inevitably lead to an increase in bad predictions and a flood of difficult-to-resolve errors. In this way, the AIF provides the theoretical tools for thinking about, and modelling, subjective wellbeing (see, e.g. Miller et al. 2022, Smith et al. 2022).

Active inference theorists are beginning to develop novel accounts of mental health pathology which focus on the predictive effectiveness of a person’s generative model. In fact, the AIF is now considered to be a leading approach in computational psychiatry. There are a wide variety of reasons why the AIF is gaining popularity in computational psychiatry, including offering a unified account of perception, learning and decision-making, and new ways of modelling ‘explore-exploit’ trade-offs. While some of the same conclusions may be drawn from simpler approaches (e.g. traditional reinforcement learning models), these often lack the computational specificity and the unifying potential that the AIF offers. For a more indepth look at what the AIF offers to computational psychiatry, see (Parr et al. 2018, Smith et al. 2021b and Constant et al. 2022). The AIF offers new (computationally grounded and neurobiologically plausible) accounts of various psychopathological conditions, such as depression, schizophrenia, depersonalization, obsessive compulsive disorder, addiction, and functional motor and sensory symptoms such as chronic pain—see, e.g. (Fletcher and Frith 2009, Seth et al. 2012, Corlett and Fletcher 2014, Seth and Friston 2016, Barrett et al. 2016, Badcock et al. 2017, Fabry 2020, Kiverstein et al. 2020, Miller et al. 2020, Deane et al. 2020, Gerrans 2022, Smith et al. 2021b, 2020b). In the following sections, we take first steps towards applying this framework to understanding some of the sub-optimal relationships that can emerge specifically between humans and technology. In particular, we will look at how some of both the inherent, and deliberately designed, features of social media platforms can pose a threat to the ongoing effectiveness of a generative model—and with it our mental health.

## Your brain on social media

Social media can act as a spectacularly effective method for warping our generative models, as it often bombards users with bad evidence about both the offline environment around us and our place in it. Typically, in the offline world, our generative model and expectations are encoded with information incoming from an unfiltered environment, which means that most of the time, our generative model more or less accurately (or at least usefully) reflects the world. However, in cases of regular and heavy engagement with certain content on social media, incoming information about the world is very often carefully selected, curated, and digitally altered—we are potentially engaging with a fantasy. Moreover, apps that offer the use of filters also allow us to represent ourselves in carefully curated ways, potentially cultivating kinds and quantities of feedback and validation simply not available to us when we go offline. The space between being and appearing is potentially vast—with a few swipes, we can dramatically alter our appearance, or retake the same picture 20 times until our face exudes precisely the calm mastery of life we want to project. As social media platforms develop features which foster an increasing potential for inauthenticity, the more those platforms become potentially powerful bad-evidence generators, flooding the cognitive machinery of their users with inaccurate information, telling us that the world is full of incredibly beautiful, cool people, living wonderfully luxurious lives: social media platforms can act as a digital crowbar, prising apart our generative model from the offline environment. Instead, our model of the ‘real’ world comes to produce the expectations generated through the online environment, and the result is, potentially, increasingly unmanageable waves of prediction error which the system must now strive to minimize. One crucial and disconcerting aspect of this picture is that ‘top-down’ knowledge is not guaranteed to protect us from these effects. We might think that we could simply remind ourselves to treat much online content as a kind of misinformation. However, research has shown that even applying explicit ‘disclaimer labels’ to altered images—labels that highlight the image as being misrepresentative of reality—has no impact at all on how those images make people feel (Tiggemann et al. 2013, 2014, 2019, Tiggemann and Brown 2018). Indeed, for most of us, the fact that online content is not representative of how the real world is, is not a revelation. Our generative models, however, are deep and absorb evidence and update expectations on an automatic and subpersonal level. Consider the fact that a placebo medication can work even though we are told beforehand that we are going to be given a placebo (Marshall 2016). It may be that our predictive systems automatically soak up the expected evidence of medical treatment—white coats, stethoscopes, pills—and update expectations in spite of higher-level knowledge about placebos.

The seemingly extreme actions of seeking cosmetic surgery to look more like one’s online presence, then, are part of just one strategy for resolving this kind of prediction error. A recent survey found that more than half of cosmetic surgeons had patients ask explicitly for procedures which would enhance their online image, while many also reported patients using enhanced images of themselves as an example of how they would like to look (Hunt 2019). One filter, mentioned by prominent social media influencers, allowed users to preview the effects of specific cosmetic procedures, and while Instagram has now banned that specific filter, many perform very similar functions. While this may seem extreme, these actions make perfect sense when viewed through the AIF. If we become accustomed to our own doctored appearance, and to receiving all of the feedback associated with it, soon the level of validation available offline will be registered as a mounting prediction error that is likely to result in feelings of stress and inadequacy. According to the AIF, seeking surgery to bring our offline self in line with our online self is no different from grabbing a blanket as the temperature begins to drop: we are sampling the world to bring us back into an expected state, acting to minimize prediction error. It is also a strategy that is becoming easier to pursue, given that cosmetic procedures are becoming cheaper, more widely available, and less stigmatized (Sincere thanks to one of our anonymous reviewers for highlighting this important point.) Under the AIF, all else being equal, agents will choose the action policy presenting the least amount of complexity and friction; reducing complexity in action is itself an effective policy for minimizing prediction error (Clark 2016). It is just that through very deliberate design features, social media is—for some users—capable of displacing our self image so much that the only way to rectify the error and meet those expectations is to surgically alter the way we look.

At this point, it is important to mention briefly (more on this later), that human beings these days inhabit many kinds of online worlds—just as real as any offline world—in which we might represent ourselves in inauthentic or misleading ways. For example, massively multiplayer online role playing games (MMORPGs) such as the highly successful World of Warcraft series, give players the opportunity to represent themselves using a diverse range of avatars, from various quasi-human figures to dragons and other creatures, and to represent themselves to others socially in online interactions. For many players, these online worlds are just as real as the offline world, replete with rich environments to explore friends, meaningful relationships, and a range of meaningful tasks. A crucial question is, then, why do those who engage regularly and heavily with MMORPGs seem to keep intact generative models when returning to their offline environments, instead of experiencing the rising prediction errors and sense of dysmorphia sometimes reported in relation to social media. To put the puzzle another way: why do the expectations associated with these MMORPGs not ‘seep’ into our offline worlds in the way that the inauthentic content of social media apparently can? We think that there is more than one answer to this question, and it is something we address in more detail later. For now though, one important reason why these worlds generally do not seep into offline life is that when we represent ourselves in this way—as a powerful elven warrior in World of Warcraft, for example - we are not implicitly suggesting to those around us online that we are in fact a powerful elven warrior in our offline life. For many users of social media platforms though, the whole point of posting content, whether digitally altered or not, is to do precisely this: to implicitly say something about their offline lives. Closely tied to this implicit claim is that the content of most social media posts is realistic (which is to say, it resembles the form and conventions of the offline world) in a way that MMORPGs typically are not.To be clear here, we are talking about the realism of the self-presentation—the features of ourselves and our lives that we are presenting to and sharing with others, rather than the realism of the mode of interaction, i.e. realtime chat through a headset versus ‘likes’ on an instagram post. Realism might play an important role, because as a consumer of content, viewing the profiles and posts of others, whether or not the ‘evidence’ generated by such content updates my own expectations about myself and the world, will depend on my prior expectations around the likelihood of that evidence being reliable. For example, if an old friend from school posts claiming to have grown wings and the ability to fly, my current generative model will assign very low prior probability to this being true. However, if my friend posts claiming to be very successful and wealthy, sharing pictures of exotic locations and a life much more luxurious and leisurely than mine, our priors for these propositions will be different. It is important that in talking about the potential pitfalls of digital technology like social media, the account we present does not merely erect a shallow dichotomy between the ‘real’ offline world and ‘fake’ online worlds. This story needs to be nuanced, to present a more detailed and subtle picture that can capture what it is about some online worlds that seem to have the potential to cause problems, while others do not.

Returning to the case of Snapchat surgery and social media, note how high the stakes are in this scenario. Surgery might offer one way to attempt to resolve the mounting error, but if we are unable to resolve the error and continue to engage with social media, then this consistent failure is fed back to the system, eventually teaching it to expect its own failure and inability to act effectively in the world. This ‘pessimistic’ tendency in prediction bears a striking resemblance to the kind of scenarios now described by neuroscientists working on computational accounts of depression based on the AIF. Various forms of psychopathology, including depression, have now been described as a form of ‘cognitive rigidity’, wherein the system fails to adjust its expectations (including expected rate of error reduction) in line with feedback from the world (Badcock et al. 2017, Kiverstein et al. 2020). While the picture of depression as a form of rigidity or inflexibility within the cognitive system is not unique to the AIF, the AIF does offer a new computational model for understanding how systems can become pathologically rigid in this way, and why that rigidity can become self-sustaining. In properly functioning predictive systems, when there is failure to resolve error in line with expectations, negatively valenced affect feeds back to the system and downregulates expectations accordingly, which then leads to the system being likely to once again resolve error in line with new expectations, which results in positive affect and an upregulation in expectation (Kiverstein et al. 2020). This constant undulation of expectation and valenced affect serves to keep well-functioning agents in a relatively stable state. In AIF accounts of depression, however, when error is not reduced in line with expectations, the system fails to update those expectations, leading to ongoing failure and an inverse slope of error reduction. The long-term summation of error manifests as persistent low mood, leading to a downregulation of precision on action policies. In short, a system which displays this rigidity in expectation comes to predict its own failure and ineffectiveness, which manifests on the subjective level as symptoms of depression, such as feelings of helplessness, isolation, lack of motivation, and an inability to find pleasure in the world (Kiverstein et al. 2020).

A 2018 exchange between Instagram user ‘ScarlettLondon’ and Twitter user ‘Nathan’, illustrates widespread intuitions about a link between social media and depression. ‘ScarlettLondon’ posted an image of her ‘morning routine’ with a caption reading ‘I…give you a little insight into how I start my day in a positive way’. The image featured Scarlett in a luxurious hotel room, with a selection of breakfast dishes laid out on the bed, complete with a product placement for Listerine. ‘Nathan’ reposted the image with another caption, reading ‘Fuck off this is anybody’s normal morning. Instagram is a ridiculous lie factory made to make us all feel inadequate’ (Moss 2018). Nathan’s sentiment captures a pervasive intuition, since attested to by several studies: that social media can cause depression because it facilitates negative comparisons with inauthentic or otherwise unattainable content (Curtis 2014). Indeed, engagement with social media platforms has been shown to have a measurable impact on an individual’s expectations of a specific place or event (Narangajavana et al. 2017). Through ongoing and consistent engagement with inauthentic content, a user’s expectations for successful error reduction in the environment have the potential to effectively be ‘pinned’ in place, leading to the predictive system being unable to flexibly adjust those expectations in the face of evidence of failure coming in from the offline world. This ongoing failure eventually teaches the system to expect failure—to predict its own inefficacy in the world—which is precisely the scenario described by AIF accounts of depression. Thus, social media can put us in a bind: either we somehow bring the world into line with our new expectations, which might involve drastic action, or we risk experiencing symptoms of depression, engendered by an influx of inaccurate evidence which renders our generative model inflexible and inaccurate.

## Designing addictive digital spaces

Of course, there is a more obvious way to alleviate any rising prediction error resulting from too much time online: spend less time online. For some of us, this is easier said than done though, as mounting evidence supports the suspicion that social media can be addictive. A comprehensive 2015 review defined social media addiction as a disproportionate concern with and drive to use social media that impairs other areas of life, and found that roughly 10% of users exhibit symptoms of addiction (Andreassen 2015). Interestingly, this is around the same percentage of people who have problems with alcohol—but while the addictive hooks of alcohol and other drugs are relatively well understood and uncontroversial, those of behavioural addictions such as engagement with social media are still subject to debate (Kardefelt-Winther et al. 2017). Some researchers argue that there is in fact no such thing as internet addiction at all (Yellowlees and Marks 2007).

Again, the AIF has the tools to help understand how engagement with these kinds of digital stimulants might lead to symptoms of addiction.

The AIF offers a new understanding of addiction as a derailment of the alignment between predictive systems and their environment (Schwartenbeck et al. 2015, Miller et al. 2020, Smith et al. 2021a, Gerrans 2022). Life contains various kinds of rewards: sex, food, status, etc, but for the brain, all that matters is reducing prediction error, bringing us closer into expected states across various timescales. Dopamine encodes and reinforces behaviours that seek and pursue prediction error reduction (Friston et al. 2012). Just like alcohol and other drugs, digital environments threaten to disrupt this balance between naturally occurring rewards and reward seeking behaviour. In his important book ‘Your Brain on Porn’, Gary Wilson argues that internet pornography presents itself as dangerously rewarding, pointing out that in one evening, internet porn facilitates levels of sexual novelty, which would have been unavailable to our ancestors across an entire lifetime: multiple tabs or windows, hundreds of different scenes and participants, escalating fetishes, all conspire to have our reward circuitry screaming ‘wow, we’re doing far better than we ever thought possible!’, when in reality, we are just staring at a screen, alone. The novelty is particularly enticing, as our brains are always seeking new ways of reducing error, novel strategies for doing better than expected (Hesp et al. 2021). Our brains register this as a huge resolution of uncertainty, and dopaminergic circuitry in the brain goes into overdrive, reinforcing and entrenching these particular reward seeking behaviours.

What pornography is to sex, social media platforms are to our intrinsic appetite for socializing. Engaging in meaningful interpersonal bonding engages all of the reward circuitry mentioned above: it feels good to socialize, and dopamine entrenches learning for successful social behaviours (Kopec et al. 2019) One major similarity between social media and pornography is that both take a naturally occuring reward (sex and social behaviour, respectively), engineer a powerful vehicle of carefully curated fantasy, and present it as an attainable and desirable reality. These presentations of ‘better than real life’ scenarios (e.g. carefully staged and filtered images; maximally exciting sexual encounters in pornography) are highly alluring for predictive agents always on the lookout for ways to improve. On social media—just as with online porn—high levels of novelty and excess mean that the reward system is kicked into overdrive. Through social media, hyperstimulation can work to reorganize our predictive model and restructure our habits: we wake up and reach for our phone, never leave home without it, and constantly feel drawn toward our phone even when in the company of friends.

One avenue of objection here might be to point out that it seems debatable to what extent social media actually captures the reality of offline social interactions, devoid as it is of many features of face to face communication, and therefore it should be unclear just how rewarding online social interactions actually are. In response to this, we can first return to the comparison with online pornography, which clearly lacks the same substantive character as real sex and relationships. Nevertheless, engagement with online porn has been shown to powerfully engage the brain’s reward seeking machinery (Negash et al. 2016; de Alarcón et al. 2019). This returns us to the point made earlier that, according to the AIF, all rewards are fundamentally processed as prediction error minimisation—crack cocaine or explicit imagery, it is all the same: the system learns to expect states where error is reduced in line with or better than expected (Miller et al. 2020). While social media certainly lacks the face to face nuance of real-world interaction, it nevertheless works hard to turbocharge many of its most gratifying aspects, such as judgement, monitoring, and validation and positive feedback.

In order to see how social media takes these rewarding aspects of social interaction and hyper charges them, first notice how all digital space has the inherent quality of dissolving the temporal and spatial restraints which govern offline interaction, thereby—in the case of social media—facilitating an excess of novelty and validation which simply is not available in the real world. Users can instantaneously exchange direct messages with people who may well be complete strangers, and when users get bored of the content they are currently interacting with, a quick swipe generates new, exciting, ad unpredictable content. These structural features—which deliberately elicit anticipatory states and facilitate near endless potential for novelty—are something that deflationary accounts of social media addiction often fail to emphasize. However, the potentially addictive nature of social media platforms does not only emerge from an excess of carefully edited content and potentially massive social feedback but also emerges from a deliberately designed and carefully implemented functional architecture which draws on our knowledge about the brain’s reward circuitry and established approaches in the gambling industry. In gambling what is so arousing (and habit-forming) is the anticipation of reward, or the expectation of an uncertain reward (Van Holst et al. 2012). Of course, offline social interactions are often unpredictable too, in that we do not know when someone might contact us or interact with us in rewarding ways, but social media sites are engineered to compound this anticipation through gamification, in which features such as progression, points scoring, and risk taking are introduced into a non-game setting. Social media gamifies social interaction, primarily through various highly interactive systems of ‘likes’, ‘shares’, ‘upvotes’, comments, and so on, which apply to user created content. This feedback is the direct measure of the ‘success’ of a particular post, and allows for comparisons in popularity between posts and posters.

When the potentially enormous levels of social feedback do come, it is not immediately communicated to the user. Rather, we receive notifications in the form of a shining button or exciting sound which delays the discovery of the precise nature of the incoming content. The simple act of pushing a button to reveal information has been shown to trigger arousal and compulsive behaviour, and newly developed features on smartphones add further layers of anticipation (Atler 2017). The ‘swipe to refresh’ feature of the Facebook app’s news feed, for example, where users physically swipe the screen to generate a new stream of information, is a startlingly similar action to the pulling of a casino slot machine arm. In each case, users do not know for sure what kind of content will spring up until they swipe. This feature, coupled with the fact that Facebook’s feed is now effectively infinite has led to the app being described as ‘behavioural cocaine’ (Andersson 2018). One final layer of anticipation comes through the use of a smartphone itself, compounding the intermittency arousal of feedback and interaction: we have been so conditioned by anticipation of smartphone buzzing that ‘phantom vibration syndrome’—the erroneous sensation of our phone vibrating—now affects 65%–89% of people who use smartphones (Rothberg et al. 2010, Drouin et al. 2012). Crucially, these carefully engineered spikes in user anticipation mirror the anticipatory states known to underlie problematic gambling; in people who exhibit addictive gambling behaviour, dopamine response has been shown to be most pronounced during phases of high anticipation (Hegarty et al. 2014). Rather than the reward itself, it is these highly arousing states of expectation of reward which have been shown to elicit the strongest dopaminergic response (Van Holst et al. 2012, Linnet 2014), and the designers of these digital platforms know this.

So far, we have focused on the way in which existing social media platforms are deliberately designed to be habit forming, to keep users ‘engaging’ even when they might be experiencing a downturn in wellbeing. We have also used the AIF to provide a theoretical account of how it is that these existing platforms cause these downturns in wellbeing in the first place. In the section that follows, we will investigate some puzzling cases: cases of interaction with digital tools and platforms that do not lead to the kinds of outcomes described earlier, and we suggest possible reasons that differentiate these cases from traditional social media platforms.

## Hope for the future

The distinction we are making here is not one as coarse-grained and clumsy as merely ‘real’ offline worlds and ‘fake’ online worlds, with online worlds always being dangerous and bad. Rather, we need a more subtle account, which understands all of these worlds—online and offline—as equally real, and that is able to provide a more nuanced account of why some of these online worlds might be a threat to the general wellbeing of predictive systems like us. Indeed, there exist a wide range of cases where the use of digital platforms, online worlds, enhanced avatars, and so on, seems to be either entirely harmless or beneficial for our wellbeing. We will mention just two.

The first case involves the use of digital manipulations to safely and cheaply explore realistic real-world options. There are many familiar examples. Those apps that let you see what you would look like if you chose a certain pair of glasses, or had a certain haircut, are a case in point. These can actually have the opposite effect to the kinds of highly filtered and idealized Instagram postings that we considered earlier. Very often, people turn up at the hairdressers with totally unrealistic expectations about how they might look if a certain celebrity hairstyle was somehow superimposed on their standard-issue face. The use of highly constrained apps can help us overcome this tendency, and facilitate real-world choice by helping us explore a range of guaranteed-realistic options. There are similar benefits available from apps that populate a photo of your kitchen or bathroom with options from a catalogue. These apps allow us to swap out and swap in new features of our bodies and environments as a kind of ‘test run’ for reality in ways that never blur the line between authenticity and inauthenticity. One way to think about this whole class of cases is as helping tune our own internal generative model in ways that deliver realistic and implementable yet mildly ‘optimistic’ predictions about how we will look. Work on the AIF already identifies as necessary a kind of ‘optimism bias’ insofar as to achieve our goals we need to predict that we will do so (Van de Cruys et al. 2020). The key to making this work is to constrain our predictions to what might be thought of as a goldilocks zone of ‘just the right amount of optimism’—enough to push us to work hard and achieve our goals. Overstep that mark and we might risk warping our generative model, as we cannot eliminate the errors and discrepancies. Understep it and we will fail to expand our abilities and achieve enough to make us content with our progress. Another crucial distinction between this class of cases and the kind of social media platforms discussed above is that these kind of apps— augmented reality which allows us to actively explore options for our offline life—is that these apps are not inherently social in the same way, and therefore lack all of the features of gamification talked about earlier. Apps like these allow us to tune our model to deliver optimistic predictions but do not then actively facilitate and encourage the presentation of these augmentations to others in ways that present that content in ways that make ambiguous the authenticity of the content. However, we should not be too quick to locate the potential harmful effect of social media platforms solely in the fact that they are social, as a look at a second class of cases will demonstrate.

The second class of cases include the highly unrealistic kind of MMORPG mentioned earlier, in which a player might choose an avatar which diverges from their offline appearance in dramatic or more fundamental fashion, such as a dragon or an orc. These cases are certainly social, and encourage the presentation and sharing of content with others, very much in a heavily gamified setting. In these cases, players are typically not tempted to attempt to alter or modify their real-world bodies to look like dragons or orcs. Why is this? Well, as discussed earlier, one crucial difference is that the worlds and characters of MMORPGs do not present as representations of real life in the same ways that many social media platforms do. But we think that there are more key differences, which might be instructive when considering exactly what might make an online platform or tool potentially harmful. The first of these key differences is that the altered self-presentations of things like MMORPGs are tethered to a distinct virtual world, a world marked by different practices and customs, and most importantly expectations, sufficiently divergent from those in the offline world—while entering a haunted crypt and slaying a troll might be a solid day of work online, it is hardly the expectation for a productive offline weekday. So, switching from one world to the other, online to offline, becomes rather like changing continents and driving on the left instead of the right. Different rules apply, and with practice, the switch becomes painless, but you never become tempted to try and alter the right-drive world to be more like the left! The second difference, closely related to this, is that the form or avatar is usually very far indeed from your daily-life form. It may be able to fly, though we are not so foolish as to seek surgery to add wings to our daily-life bodies. These two differences act as a kind of ‘cognitive immunisation’ against the kinds of damaging ‘seepage’, from the digital realm to daily life that we have been attempting to explain and diagnose.

This notion of ‘seepage’, then, might be a good place to start in terms of thinking about how to design online digital technology that prevents our generative models of the offline world becoming warped in ways that lead to harm. We use seepage to refer to the way our prior expectations about how the world should be, and how we should be in it—expectations that affect how we perceive and experience the world—can be influenced through interactions with digital technologies such as the online worlds of social media and MMORPGs; Recall that according to the PP framework, our predictions are based on sets of prior expectations about the environment.

Of course, there are lots of things that can influence our prior expectations—our models are wonderfully flexible and sensitively tuned to new evidence (it would be pathological to not exhibit this sensitivity to new evidence), but if the priors that make up our generative model come to be influenced significantly by engagement with an online world such that our ability to effectively predict and minimize prediction error offline is hampered, then we can say that one world has seeped into another. In comparing the different classes of cases in terms of online digital worlds, and looking at how engaging with these technologies impacts our experience of the offline world, we can work toward a principled approach to reducing seepage from one world to the other. For instance, working from the cases mentioned above, including social media, we can say that one factor affecting seepage seems to be how close of a resemblance one world bears to the other; MMORPGs are greatly divorced from the offline world when compared to the filtered world of Instagram, which also presents itself as representative of the offline world in a way that things like MMORPGs do not. In this way then, there might be a kind of ‘uncanny valley’ of seepage, wherein it is only when online worlds are realistic, and presented as such, that we risk seepage.

Another factor that we think is likely to be highly significant, is how some content is hyper-idealized in a way that plays into powerful social pressures and expectations, and is therefore highly salient for our sense of self and self-esteem. For example, existing insecurities within our self models might leave us more vulnerable to seepage of certain kinds of expectations. If I am insecure about my body, then being bombarded with images of people with ‘perfect’ bodies, alongside the usual and well-ingrained social pressures around bodies, might conspire to affect us in ways that the very unrealistic images within most MMORPGs do not. There is little social pressure to be a 10-feet tall orc, and so our self models might be far less vulnerable to that kind of content. Following this line of thought, we could recognize that existing models of social anxiety disorder could support the hypothesis that harmful seepage is more likely in cases where an individual already experiences engendered negative expectations around the self and social outcomes. Social anxiety disorder patients exhibit a strong overdependence on positive social feedback (Gilboa-Schechtman et al. 2000, Weisman et al. 2011, Aderka et al. 2012), and a reduced ability to handle the uncertainty associated with social interaction (Boelen and Reijntjes 2009, Carleton et al. 2010, Campbell-Sills et al. 2011, Carleton 2012). Some social media platforms provide an online world of social affordances that seem tailor made for these symptoms: massive levels of feedback and validation, coupled with an increased sense of control through the curation of an online presence. If this line of thinking is correct, then the danger is obvious: a vicious feedback loop in which increased engagement with social media, through updated expectations, further reduces the individual’s capacity to deal with offline uncertainty and the lack of constant validation and positive feedback, thereby increasing the likelihood of further engagement.

Fully understanding how we are vulnerable to expectations from some online worlds seeping into our model of the offline world is an important area of future research. However, in the remainder of this paper, we will discuss some possible avenues for reducing any potentially harmful impacts of seepage. One way forward would be to limit what kinds of filters are available to use on what kinds of platforms. For example, on highly social platforms, with features of gamification such as shares and likes etc., it might be beneficial for us to only use filters that make major and dramatic changes. Some filters on Instagram are already like this—filters that replace users’ faces with the face of a lion or other animal are common, and generally do not leave users feeling awful about not being a lion. Alternatively, we could limit filters to the kind of augmented reality apps mentioned earlier that makes only minor tweaks such as replacing one haircut with another. Filters like the ones available now that allow us to dishonestly misrepresent ourselves through airbrushing and enhancement could be limited to non-social platforms. A closely related and intuitively plausible solution would be to require all filtered, or digitally altered content to be flagged, or labelled, thereby alerting the viewer to the fact that they are in fact not viewing content which should be taken as representative of the offline world. However, as mentioned earlier, several studies have shown that this is ineffective and can even make people feel worse about themselves after interacting with flagged images (Tiggemann et al. 2014, Tiggemann and Brown 2018).

However, issues with seepage and the question of what and how to legislate go beyond images. Tufekci (2017) gives a disturbing and striking example of seepage; the case of Michael Brutsch, a man involved in a ‘community’ of users of the website ‘Reddit’, who gathered to share sexualized images of underage children. When this group gained attention, Brutsch appeared on mainstream television in an unsettling interview, during which he seemed incapable of understanding that what he had done was wrong, consistently referring to the mechanics of the website to normalize his behaviour. Tufekci writes:

He still seemed stuck between two sets of norms, those of the community of Redditors to which he had belonged for so long, and the mainstream norms he was now facing…Brutsch was clearly startled by this confrontation between the distorted norms his community had built and those of the rest of the world (pp. 169).

In her analysis of this case, Tufekci suggests that specific design decisions regarding the Reddit platform had a considerable impact on what happened—decisions such as the allowing of pseudonymity, and gamification through ‘upvotes’ and ‘karma points’. These features, she suggests, contributed to a strong sense of community and validation which seeped into Brutsch’s model of the offline world, meaning he struggled to switch gears when being interviewed within the context of offline norms and expectations. Thus, it may also be the case that features such as these turn out to be something we might want consider when designing our online worlds. Identifying the variables associated with seepage and seeking to control them through legislation might work but is likely to be difficult to implement and is ultimately undesirable. The better option, we think, is to recognize that we have come a long way in recognizing and responding to the many known biases in human reasoning that predate the digital age (see e.g. Kahneman 2011), and that the time is ripe to expand that palette in ways that engage the many new pitfalls and fallacies ushered onto the stage by digital platforms and social media. The idea would be to engender, through education, an awareness of the kinds of pitfalls, and weaknesses discussed in this paper. This kind of education would, we think, be part of a broader project that is a kind of ‘epistemology for the digital age’. This is, of course, not an entirely new suggestion. Both Shannon Vallor’s work on technomoral virtues and Zeynep Tufekci’s work have highlighted a need for us to be sensitive to the kinds of affordances provided by digital technology, and how these affordances might pose challenges for us. We think that the PP framework is well poised to help build a clearer and more helpful picture of how digital affordances have the kinds of effects they do.

## Conclusion

The AIF has, in recent years, come to change how we understand a range of psychological phenomena, including addiction and depression. In this paper, we have used the theoretical tools of active inference to enter into an ongoing debate about the ways in which social media—and digital environments more broadly—have the potential to negatively impact our mental wellbeing. While deflationary accounts downplay the effects of the design and structural features of digital environments, this active inference account adds weight to arguments that there are engineered features of digital technology that can have profound consequences for our wellbeing. These arguments may have a wide ranging impact, given that these inherent features are deliberately implemented. As design guru Nir Eyal states, ‘Companies increasingly find that their economic value is a function of the strength of the habits they create’ (Eyal 2014). As it turns out then, the designers of social media, aiming to maximize engagement through design, may have a *de facto* interest in increasing the corrosive effect their platforms have on the mental health of users. Seen in this context, this emerging picture may lend significant weight to arguments that we should take digital hyperstimulants seriously as a threat to our wellbeing, and to voices calling for changes to the way digital technology like social media is designed, operated, and regulated.
